# Outcomes among patients undergoing transcatheter aortic valve replacement with very low baseline gradients

**DOI:** 10.3389/fcvm.2023.1194360

**Published:** 2023-08-04

**Authors:** Faisal Rahman, Hetal H. Mehta, Jon R. Resar, Rani K. Hasan, Wendy Marconi, Hamza Aziz, Matthew J. Czarny

**Affiliations:** ^1^ Division of Cardiology, Johns Hopkins Hospital, Baltimore, MD, United States; ^2^ Division of Cardiology, Doylestown Health, Doylestown, PA, United States; ^3^ Division of Cardiac Surgery, Johns Hopkins Hospital, Baltimore, MD, United States

**Keywords:** low-gradient aortic stenosis, transcatheter aorta valve replacement, patient outcomes, quality of life, valvular heart disease (VHD)

## Abstract

**Background:**

While there is evidence that patients with low-flow, low-gradient aortic stenosis (AS) benefit from transcatheter aortic valve replacement (TAVR), data are lacking regarding outcomes of patients with a very low gradient (VLG).

**Methods:**

In this retrospective, single-center study of patients with severe AS who underwent TAVR, three groups were defined using baseline mean aortic valve gradient: VLG (≤25 mmHg), low gradient (LG, 26–39 mmHg), and high gradient (HG, ≥40 mmHg). The primary outcome was the composite of Kansas City Cardiomyopathy Questionnaire (KCCQ)-12 of <45, decrease in KCCQ-12 of ≥10 compared with baseline, or death at 1 year.

**Results:**

One-thousand six patients were included: 571 HG, 353 LG, and 82 VLG. The median age was 82.1 years [interquartile range (IQR) 76.3–86.9]; VLG patients had more baseline comorbidities compared with the other groups. The primary outcome was highest at 1 year in the VLG group (VLG, 46.7%; LG, 29.9%; HG, 23.1%; *p* = 0.002), with no difference between groups after adjustment for baseline characteristics. At baseline, <30% of VLG patients had an excellent or good (50–100) KCCQ-12, whereas more than 75% and 50% had an excellent or good KCCQ-12 at 30-day and 1-year follow-up, respectively.

**Conclusion:**

Although patients with VLG undergoing TAVR have a higher rate of poor outcomes at 1 year compared with patients with LG and HG severe AS, this difference is largely attributable to baseline comorbidities. Patients with severe AS undergoing TAVR have significant improvement in health status outcomes regardless of resting mean gradient.

## Introduction

Aortic stenosis (AS) is a common valvular heart disease in the developed world, and medically managed patients with symptomatic severe AS have up to 50% mortality at 2 years (1). Severe AS has historically been defined by a peak aortic transvalvular velocity of ≥4 m/s or a mean gradient of ≥40 mmHg, with an estimated aortic valve (AV) area of ≤1.0 cm^2^. However, it has become clear that while these parameters define “classical” high-gradient severe AS, it is possible to have severe AS not meeting these traditional parameters. Because transvalvular velocity and gradient are determined by both AV area and transvalvular flow, severe AS may be present despite a peak velocity of <4.0 m/s and a mean gradient of <40 mmHg if transvalvular flow is reduced. This entity, known as “low-flow, low-gradient” severe AS, can exist in patients with both preserved and reduced left ventricular ejection fraction (LVEF, known as “paradoxical” and “classical” low-flow, low-gradient AS, respectively) and is defined as an AV area of ≤1.0 cm^2^ with a peak velocity of <4.0 m/s, a mean gradient of <40 mmHg, and a stroke volume index of ≤35 ml/m^2^ (2–5). Patients in all of these groups with true severe AS benefit from aortic valve replacement (AVR), but outcomes after AVR are strongly dependent on the resting mean gradient prior to AVR (4). However, studies of “low-flow, low-gradient” severe AS have mainly included patients with a mean gradient of >25 mmHg. Thus, patients with severe AS and a mean gradient of ≤25 mmHg is not a well-studied population with very limited data characterizing the phenotype and outcomes. This group may represent a distinct population of very low-gradient severe AS patients at even higher risk of adverse outcomes, possibly due to more advanced disease, worse left ventricular systolic and/or diastolic function, a systemic low-flow state, or a longer preceding duration of physical debilitation, and therefore may not benefit from AVR. Therefore, in this report, we aim to describe these “very low-gradient” severe AS patients who underwent TAVR and compare their outcomes to patients with high-gradient (≥40 mmHg) and low-gradient (26–39 mmHg) severe AS undergoing TAVR.

## Methods

### Study design

We performed a retrospective analysis of all commercial TAVR procedures performed at our institution from 1 January 2011 to 31 December 2020. Data were abstracted from patient charts by a trained data abstractor as part of the programmatic requirement for participation in the Society of Thoracic Surgeons/American College of Cardiology Transcatheter Valve Therapy (STS/ACC TVT) Registry, and this was supplemented by a chart review by two study authors (FR and WM). We included all patients undergoing commercial TAVR (i.e., not as part of a research study) primarily for severe AS and excluded patients with aortic insufficiency, mixed valve disease (AS with at least 3+ aortic regurgitation), or a failed bioprosthetic AV. The study protocol was approved by the Johns Hopkins Medicine Institutional Review Board with a waiver of informed consent.

The diagnosis of severe AS utilized contemporaneous ACC/AHA guidelines at the time of evaluation and included a review of all pertinent and available clinical information. In particular, the diagnosis of low-flow, low-gradient AS was made by integrating the results of diagnostic testing (potentially including dobutamine stress echocardiography and/or AV calcium scoring by computed tomography) with the exclusion of alternative causes of the patient's symptoms. Both the diagnosis of severe AS and the decision to perform TAVR were made by our institution's multidisciplinary Heart Team, which consisted of at least three interventional cardiologists trained in structural heart disease and two cardiac surgeons. Therefore, patients without severe AS or not thought to be likely to benefit from TAVR by Heart Team evaluation were excluded. Patients were separated into three groups according to the resting aortic mean transvalvular gradient derived from transthoracic echocardiography (TTE): (1) very low gradient (VLG, ≤25 mmHg), (2) low gradient (LG, 26–39 mmHg), and (3) high gradient (HG, ≥40 mmHg).

### Study procedures

Patients were clinically evaluated at baseline, 1 month, and 1 year post-TAVR with a clinic visit, TTE, and health status assessment utilizing the 12-question self-administered Kansas City Cardiomyopathy Questionnaire (KCCQ-12) (6) and the New York Heart Association (NYHA) functional classification. Other follow-up was obtained through medical record review, phone call, or electronic notification of patient admissions or discharges. All patients had baseline recording of demographic data, procedural details, and comorbidities collected by review of the medical record including notes generated during pre-TAVR evaluation. Estimated glomerular filtration rate was calculated by the Chronic Kidney Disease Epidemiology Collaboration (CKD-EPI) equation (7). Echocardiography was clinically indicated, and data were abstracted from the clinical reports. LVEF of ≥50% on TTE was defined as normal. Baseline covariates are defined in the STS/ACC TVT Registry for TAVR, version 2.0 (8).

### Outcomes and hemodynamics

The primary outcome was the composite of all-cause death, KCCQ-12 of <45, or a decrease in KCCQ-12 of ≥10 at 1 year compared with baseline. This combined outcome was previously evaluated in patients with TAVR (2). Because it combines both mortality and quality of life measures into a single composite endpoint, it serves as an important measure to assess the benefit of TAVR. Secondary outcomes included the 30-day primary outcome, 30-day and 1-year KCCQ-12, change in KCCQ-12 of ≥5 compared with baseline, mean AV gradient, LVEF, all-cause death, the composite of death or readmission, and NYHA functional classification. Time windows for 30-day and 1-year follow-up were defined by the STS/ACC TVT Registry (25–75 days post-TAVR for 30-day follow-up and 305–425 days post-TAVR for 1 year) (8).

We also performed a secondary hypothesis-generating analysis evaluating invasive hemodynamics among patients with VLG AS. Twenty-nine of the 82 patients in the VLG group underwent clinically indicated right and left heart catheterization for invasive measurement of mean gradient, measurement/estimation of cardiac output by thermodilution, and AV area estimation by the Gorlin equation (9).

### Statistical analysis

We used standard descriptive characteristics to characterize each group. Baseline demographics, comorbidities, procedural characteristics, and outcomes were compared using Chi-square tests for categorical variables and the Kruskal–Wallis test for continuous variables. Analyses of 30-day and 1-year readmissions and mortality utilized time to event analyses by Kaplan–Meier estimation and the log-rank test for comparisons. Multivariable logistic regression was used to determine the effect of gradient group on dichotomous outcomes after adjustment for differences in baseline covariates; covariates were selected for their *a priori* relevance (age, sex, race, LVEF) and for their association with the outcomes on univariable analyses (body surface area, estimated GFR, baseline KCCQ-12 score, history of atrial fibrillation or flutter, chronic lung disease, home oxygen use, dialysis, at least moderate mitral regurgitation at baseline, at least moderate tricuspid regurgitation at baseline, pre-TAVR STS mortality risk, TAVR access site, TAVR anesthesia type, TAVR procedure success, and cardiopulmonary bypass during TAVR). Cox proportional hazard models with the same covariates were used to determine the association of gradient group with time-to-event outcomes. Statistical analyses were performed using Stata version 15.1 (StataCorp, College Station, TX, USA). For missing data, we performed multiple imputation by chained equations with 10 imputations to reduce the bias of estimates of outcomes. We used the “mi estimate” command in Stata which combines the multiple imputed data sets and adjusts coefficients and standard errors for the variability between imputations based on the rules by Rubin (10). Statistical significance was defined as a two-sided α = 0.05.

## Results

### Baseline characteristics

**Figure 1:**
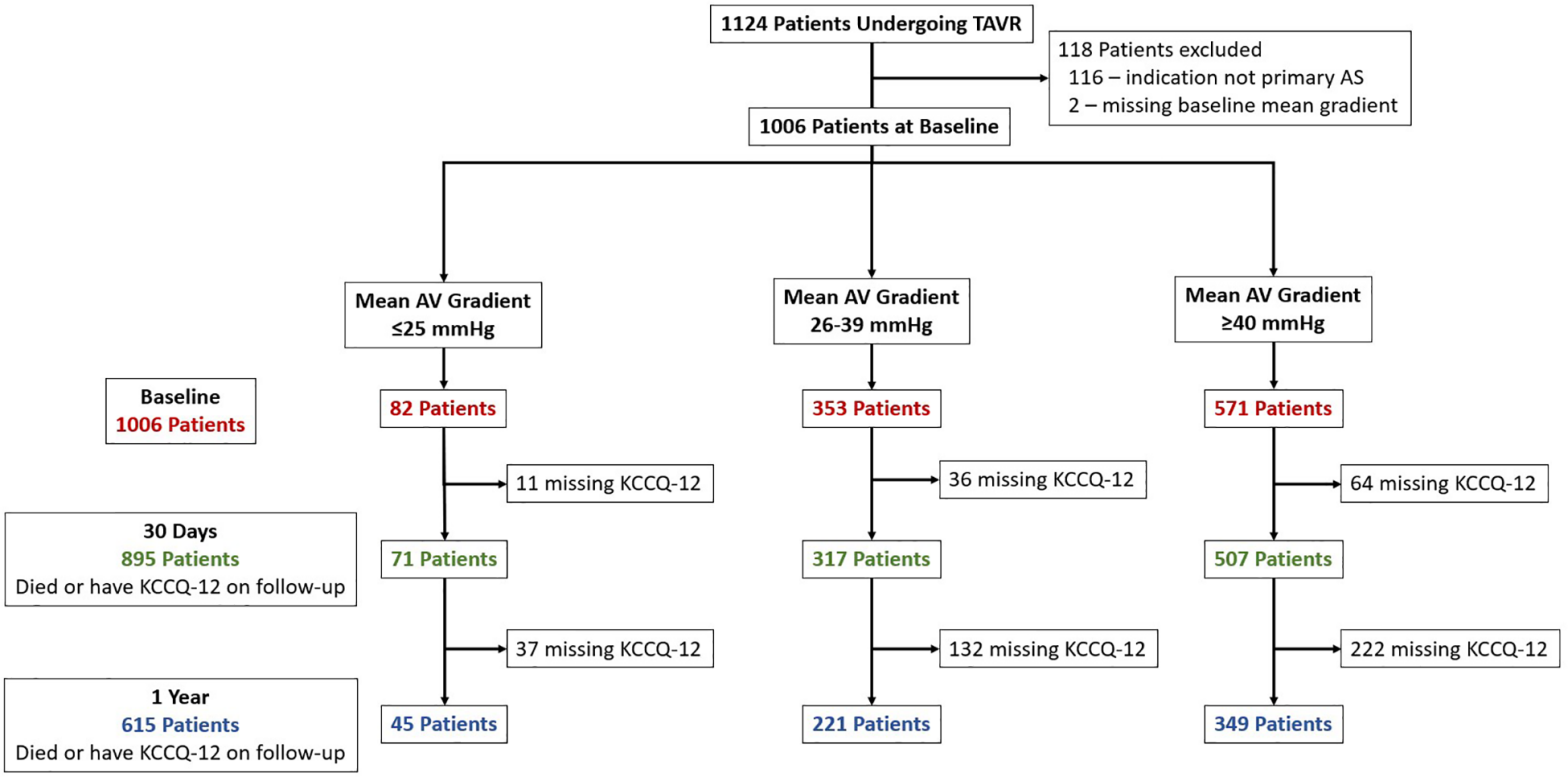
Study inclusion flowchart. Patients who underwent TAVR were identified and included if they had primary severe aortic stenosis. Patients were divided into three groups, very low (VLG), low (LG), and high (HG) mean gradient aortic stenosis, and then followed up at 30 days and 1 year. The number of patients with missing KCCQ-12 at 30-day and 1-year follow-up is shown.

Of the 1,124 patients undergoing commercial TAVR at our institution during period of analysis, 116 (10.3%) were excluded because the indication for TAVR was not primary AS, and 2 (0.2%) were excluded for a missing baseline mean AV gradient. Of the 1,006 remaining cases, 571 (57%) had HG, 353 (35%) had LG, and 82 (8%) had VLG severe AS (Figure 1). At 1-year follow-up, 615 patients (61%) either had a KCCQ-12 recorded or had died, with no significant difference between groups (*p* = 0.43). Baseline demographics and characteristics are summarized in Table 1. Compared with LG and HG groups, patients in the VLG group were less frequently female and had a higher prevalence of comorbidities including atrial fibrillation/flutter, moderate to severe mitral and/or tricuspid regurgitation, home oxygen dependence, and LVEF of <50%. Despite the higher rate of home oxygen therapy, patients with VLG had a lower rate of chronic lung disease compared with HG and LG patients. At baseline, patients in the VLG group also more frequently had NYHA class III–IV symptoms and a lower median KCCQ-12 score. The indexed AV area was slightly higher in VLG patients [median 0.41 cm^2^/m^2^, interquartile range (IQR) 0.36–0.48 cm^2^/m^2^] than in LG (0.39 cm^2^/m^2^, IQR 0.33–0.46 cm^2^/m^2^) and HG (0.36 cm^2^/m^2^, IQR 0.30–0.42 cm^2^/m^2^; *p* < 0.001) patients.

**Table 1:** Baseline characteristics according to resting pre-TAVR mean gradient.

	Overall	≤25 mmHg	≥26–39 mmHg	≥40 mmHg	*p*-Value
	(*n* = 1,006)	(*n* = 82)	(*n* = 353)	(*n* = 571)	
Age (years)	82.1 (76.3–86.9)	82.6 (78.0–87.3)	81.9 (76.1–86.9)	82.1 (76.1–86.9)	0.62
Female	48.9	41.5	41.9	54.3	<0.001
Race					
White	88.7	89.0	90.1	87.7	0.58
Black	9.3	7.3	8.2	10.3	
Other	2.0	3.7	1.7	1.9	
Hispanic ethnicity (*n* = 1,003)	1.4	3.7	0.9	1.4	0.15
BMI (kg/m^2^, *n* = 1,004)	27.4 (23.7–31.8)	26.5 (23.1–31.0)	27.4 (23.6–31.9)	27.6 (23.9–32.0)	0.073
BSA (m^2^, *n* = 1,004)	1.90 (1.71–2.09)	1.88 (1.71–2.02)	1.90 (1.71–2.10)	1.91 (1.72–2.09)	0.64
Estimated GFR (ml/min/1.73 m^2^; *n* = 1,005)	58.0 (43.3–76.0)	51.2 (38.8–69.6)	56.9 (40.8–72.7)	59.5 (45.3–78.1)	0.010
Current dialysis	3.2	4.9	2.6	3.3	0.53
Atrial fibrillation/flutter (*n* = 1,005)	39.4	58.5	48.9	30.8	<0.001
Diabetes mellitus	36.1	32.9	38.2	35.2	0.53
Chronic lung disease (*n* = 988)	52.1	45.0	57.3	49.9	0.039
Home oxygen therapy	9.2	17.1	8.5	8.4	0.034
Prior PCI/CABG	42.5	51.2	52.7	35.0	<0.001
Prior stroke/TIA	17.1	15.9	20.7	15.1	0.084
STS risk score (*n* = 991)	4.9 (3.1–8.0)	5.0 (3.7–9.7)	5.2 (3.4–8.5)	4.6 (3.0–7.7)	0.004
NYHA class (*n* = 1,002)					
I	1.1	1.2	0.3	1.6	0.010
II	42.4	28.4	40.7	45.4	
III	47.8	54.3	51.3	44.7	
IV	8.9	16.1	7.7	8.3	
KCCQ-12 (*n* = 983)	41.7 (25.5–60.4)	32.1 (17.7–52.1)	39.3 (27.1–58.3)	44.3 (26.0–64.1)	0.003
KCCQ-12 <45 (*n* = 983)	55.8	65.9	59.3	52.1	0.017
Baseline echocardiogram					
AVA (*n* = 992)	0.70 (0.60–0.84)	0.80 (0.68–0.90)	0.75 (0.62–0.88)	0.70 (0.56–0.80)	<0.001
Indexed AVA (*n* = 990)	0.38 (0.31–0.44)	0.41 (0.36–0.48)	0.39 (0.33–0.46)	0.36 (0.30–0.42)	<0.001
AV peak velocity (m/s; *n* = 956)	4.1 (3.6–4.4)	3.1 (2.9–3.3)	3.6 (3.4–3.9)	4.4 (4.1–4.7)	<0.001
Mean AV gradient (mmHg)	41 (32–50)	23 (21–24)	33 (29–36)	48 (43–57)	<0.001
Moderate or greater aortic regurgitation (*n* = 987)	6.4	3.7	6.7	6.6	0.59
LVEF (%)	63 (53–65)	55 (40–63)	58 (45–63)	63 (58–68)	<0.001
Normal LVEF (≥50%)	79.4	62.2	72.2	86.3	<0.001
Moderate or greater mitral regurgitation (*n* = 960)	23.0	30.5	26.7	19.7	0.015
Moderate or greater tricuspid regurgitation (*n* = 996)	15.5	22.2	18.8	12.4	0.007
Procedure details					
Successful procedure (*n* = 1,004)	99.1	100.0	99.2	99.0	0.64
Anesthesia type (*n* = 1,003)					
Moderate	75.9	82.9	73.3	76.5	0.16
General	24.1	17.1	26.7	23.5	
Access site (*n* = 1,004)					
Iliofemoral	95.9	93.8	94.0	97.4	0.029
Non-iliofemoral	4.1	6.2	6.0	2.6	
Cardiopulmonary bypass (*n* = 1,005)	2.1	1.2	2.6	1.9	0.69

Values are median (IQR) or %. KCCQ, Kansas City Cardiomyopathy Questionnaire; NYHA, New York Heart Association; LV, left ventricular; PCI, percutaneous coronary intervention; TIA, transient ischemic attack; GFR, glomerular filtration rate.

### Clinical outcomes

The primary combined poor outcome more frequently occurred in the VLG group at 30 days (25.4%) than in patients with LG and HG AS (19.2% and 12.8%, respectively; *p* = 0.004; Table 2). The same was true at 1 year (VLG, 46.7%; LG, 29.9%; HG, 23.2%; *p* = 0.002; Table 3). After adjustment for baseline characteristics, the VLG group had similar odds of the combined poor outcome at 30 days (OR 1.9, 95% CI 0.9–3.8, *p* = 0.074 compared with HG) and 1 year (OR 1.80, 95% CI 0.84–3.87, *p* = 0.13 for VLG vs. HG; Table 4). After imputation for missing values of baseline covariates and outcomes, VLG was associated with the combined poor outcome at 30 days (OR 2.1, 95% CI 1.1–4.1, *p* = 0.026) but not at 1 year (OR 1.8, 95% CI 0.8–3.8, *p* = 0.15; Table 4).

**Table 2:** Thirty-day outcomes according to baseline resting mean aortic valve gradient.

	≤25 mmHg		26–39 mmHg		≥40 mmHg		*p*-Value
	*N*	* *	*N*	* *	*N*	* *	
Combined poor outcome—*n* (%)	71	18 (25.4)	317	61 (19.2)	507	65 (12.8)	0.004
All-cause mortality—*n* (%)	82	5 (6.1)	353	16 (4.5)	571	21 (3.7)	0.54
KCCQ-12^a^	66	81.8 (60.4–94.8)	303	85.4 (63.5–94.8)	490	87.5 (70.3–96.9)	0.061
Change in KCCQ-12^a^	66	37.7 (15.6–56.9)	299	33.3 (13.5–53.1)	479	34.7 (14.6–53.7)	0.60
Change in KCCQ-12 Groups—*n* (%)							
≤−5		3 (4.6)		23 (7.7)		26 (5.4)	0.50
−5–5		3 (4.6)		17 (5.7)		36 (7.5)	
≥5		60 (90.6)		259 (86.6)		417 (87.1)	
NYHA class—*n* (%)	62		287		479		
I		49 (79.0)		239 (83.3)		399 (83.3)	0.20
II		8 (12.9)		41 (14.3)		65 (13.6)	
III		4 (6.5)		7 (2.4)		14 (2.9)	
IV		1 (1.6)		0 (0.0)		1 (0.2)	
Echocardiogram							
AV mean gradient (mmHg)^a^	55	6 (4–8)	286	7 (5–10)	454	8 (6–11)	<0.001
LVEF (%)^a^	55	58 (43–63)	288	60 (48–65)	458	63 (58–68)	<0.001

^a^ Values are median (interquartile range). Change in KCCQ-12 is compared with baseline. AV, aortic valve, KCCQ, Kansas City Cardiomyopathy Questionnaire; NYHA, New York Heart Association.

**Table 3:** One-year outcomes according to baseline resting aortic valve mean gradient.

	<25 mmHg		26–39 mmHg		≥40 mmHg		*p*-Value
	*N*		*N*		*N*		
Combined poor outcome—*n* (%)	45	21 (46.7)	221	66 (29.9)	349	81 (23.2)	0.002
All-cause mortality—*n* (%)	82	19 (23.2)	353	47 (13.3)	571	52 (9.1)	0.001
KCCQ-12^a^	26	89.3 (63.5–96.9)	174	88.9 (68.8–96.9)	297	88.9 (70.8–96.9)	0.86
Change in KCCQ-12^a^	26	41.9 (29.7–57.5)	167	39.6 (18.8–55.6)	286	36.7 (17.7–55.2)	0.41
Change in KCCQ-12 groups—*n* (%)							
≤−5		0 (0.0)		12 (7.2)		16 (5.6)	0.59
−5–5		1 (3.9)		8 (4.8)		18 (6.3)	
≥5		25 (96.2)		147 (88.0)		252 (88.1)	
NYHA class—*n* (%)	25		159		283		
I		18 (72.0)		131 (82.4)		234 (82.7)	0.29
II		5 (20.0)		22 (13.8)		44 (15.6)	
III		2 (8.0)		6 (3.8)		5 (1.8)	
IV		0 (0.0)		0 (0.0)		0 (0.0)	
Echocardiogram^a^							
AV mean gradient (mmHg)	30	7 (4–9)	176	8 (6–12)	289	9 (6–12)	0.002
LVEF (%)	31	58 (45–63)	180	58 (53–63)	296	63 (58–68)	<0.001

^a^ Values are median (interquartile range). Change in KCCQ-12 is compared with baseline. AV, aortic valve, KCCQ, Kansas City Cardiomyopathy Questionnaire; NYHA, New York Heart Association.

**Table 4:** Univariable and multivariable combined outcome at 30-day and 1-year by mean gradient group.

	30-day combined poor outcome^a^			1-year combined poor outcome^a^		
	Odds ratio	95% CI	*P*-value	Odds ratio	95% CI	*P*-value
Univariable analysis						
≤25	2.3	1.3–4.2	0.006	2.9	1.5–5.5	0.001
26–39	1.6	1.1–2.4	0.013	1.4	1.0–2.1	0.077
≥40	Ref	–	–	Ref	–	–
Multivariable analysis						
≤25	1.9	0.9–3.8	0.074	2.0	0.9–4.2	0.09
26–39	1.3	0.8–2.1	0.22	1.1	0.7–1.8	0.78
≥40	Ref	–	–	Ref	–	–
Multivariable analysis with multiple imputation						
≤25	2.1	1.1–4.1	0.026	1.8	0.8–3.8	0.15
26–39	1.6	1.0–2.4	0.04	0.9	0.6–1.5	0.69
≥40	Ref	–	–	Ref	–	–

^a^ Combined poor outcome defined as death, Kansas City Cardiomyopathy Questionnaire (KCCQ) score of <45, or decrease in KCCQ of ≥10. For the multivariable analysis, *n* = 803 (of 895 with outcome data) for 30 days and *n* = 540 (of 615 with outcome data) for 1 year. All patients (1,006) were included in the multivariable analysis with multiple imputation.

**Figure 2:**
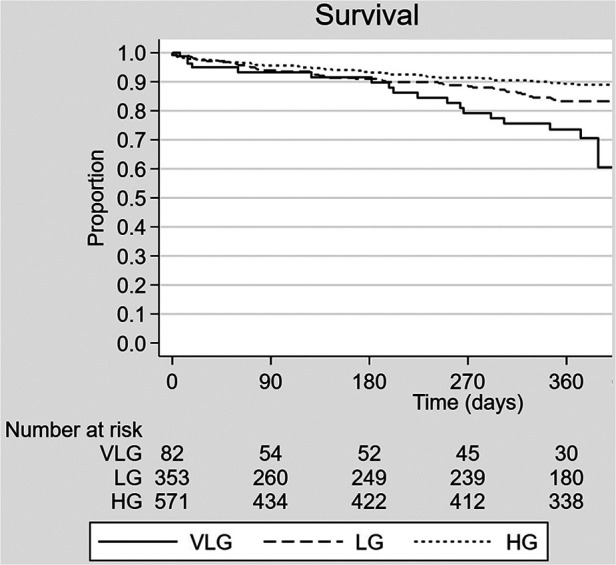
Kaplan–Meier survival curve estimates according to baseline mean aortic valve gradient group. VLG, very low gradient; LG, low gradient; HG, high gradient.

Crude mortality was similar among groups at 30 days but higher in the VLG group at 1 year, and VLG patients had the highest mortality rate over the entirety of follow-up by Kaplan–Meier estimation (*p* < 0.001 by log-rank test; Figure 2, Tables 2, 3). By Cox proportional hazards regression, VLG at baseline did not predict all-cause mortality after adjustment for differences in baseline characteristics [hazard ratio (HR) 1.8, 95% CI 1.0–3.3, *p* = 0.065 vs. HG]. No difference in cause of death through 1 year was noted according to baseline gradient group (Table 5). The composite of death or readmission was significantly less common in the HG group compared with the other two groups (Supplementary Figure S1, *p* = 0.009 for log-rank test), though again this difference did not persist after adjustment for baseline characteristics (HR 1.1, 95% CI 0.7–1.6, *p* = 0.67 for VLG, and HR 1.1, 95% CI 0.9–1.4, *p* = 0.37 for LG compared with HG).

**Table 5:** Cause of death through 1-year follow-up according to baseline mean aortic valve gradient.

Cause of death	All	≤25 mmHg	26–39 mmHg	≥40 mmHg
	(*n* = 118)	(*n* = 19)	(*n* = 47)	(*n* = 52)
Cardiac	26 (22)	4 (21)	10 (21)	12 (23)
Neurologic	9 (8)	2 (11)	5 (9)	3 (6)
Renal	2 (2)	0 (0)	2 (4)	0 (0)
Vascular	4 (3)	1 (5)	1 (2)	2 (4)
Infection	13 (11)	3 (16)	2 (4)	8 (15)
Valvular	0 (0)	0 (0)	0 (0)	0 (0)
Pulmonary	18 (15)	3 (16)	11 (23)	4 (8)
Unknown	34 (29)	5 (26)	14 (30)	15 (29)
Other	12 (10)	1 (5)	3 (6)	8 (15)

All numbers are *n* (%). *p* = 0.46 for comparison by Chi-square test.

**Figure 3:**
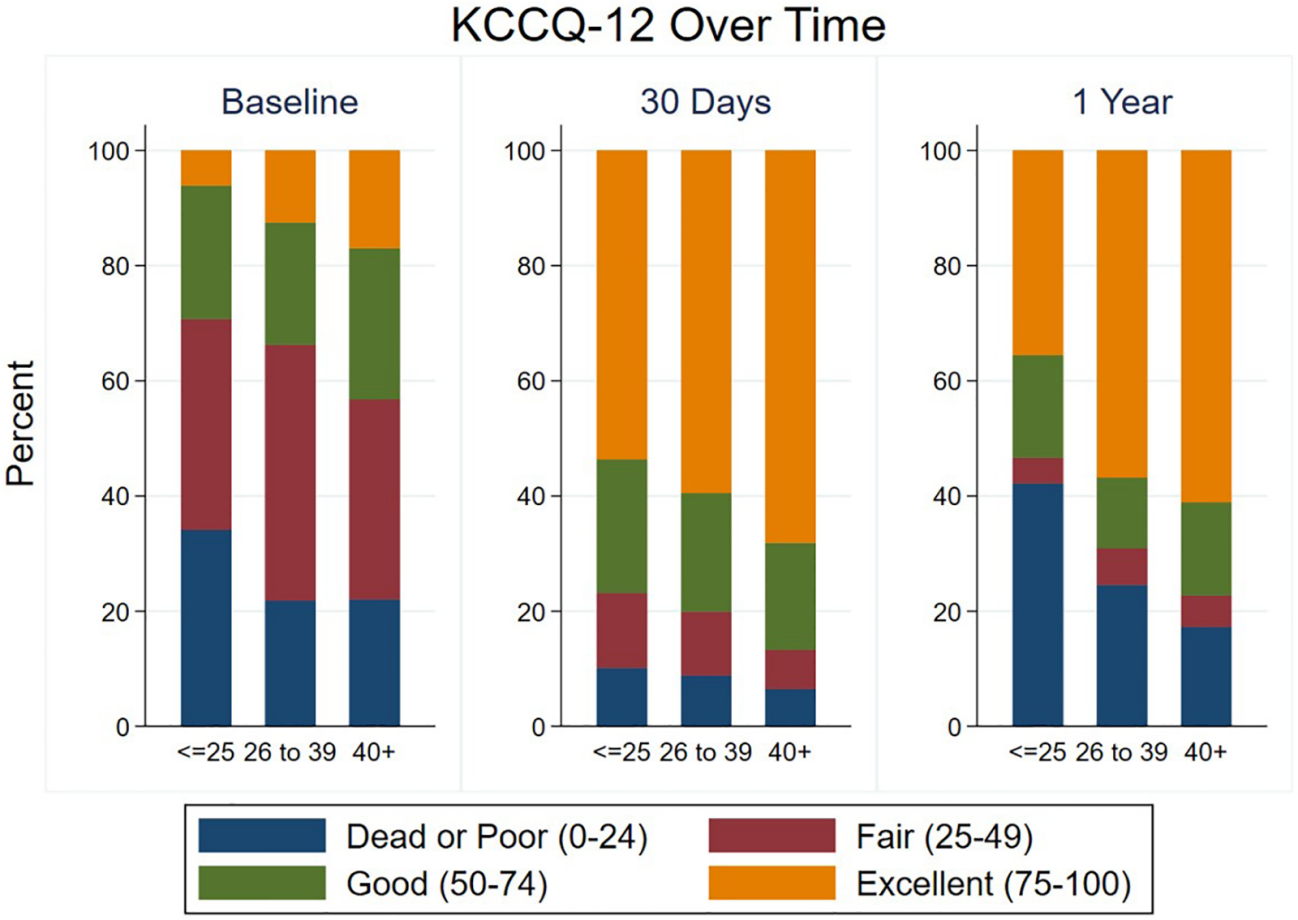
Health status at baseline and follow-up according to mean gradient group. KCCQ-12 scores are shown at baseline, 30 days, and 1 year in each mean gradient group.

Most patients had a significant improvement in their KCCQ-12, as defined by an increase of ≥5, both at 30-day and 1-year follow-up (Tables 2, 3), with no significant difference between groups at either time point. The median improvement in KCCQ-12 score was more than 30 in all groups at both 30 days and 1 year, with no difference between groups. At baseline, less than 30% of VLG patients had an excellent or good KCCQ-12 score (50–100), whereas more than 75% and 50% had an excellent or good KCCQ-12 score at 30 days and 1 year, respectively (Figure 3).

Patients in the VLG group had a lower AV mean gradient at 30 days compared with the LG and HG groups (6 mmHg, IQR 4–8 mmHg vs. 7 mmHg, IQR 5–10 mmHg and 8 mmHg, IQR 6–11 mmHg, respectively; *p* < 0.001; Table 2), and this difference persisted at 1 year (Table 3). Similarly, those in the VLG group had a lower LVEF at both 30 days and 1 year (58%, IQR 45%–63% for VLG at both time points; Tables 2, 3).

The results of the hypothesis-generating invasive hemodynamics are presented in the Supplementary Material and summarized in Supplementary Figure S2.

## Discussion

To the best of our knowledge, this is the first report to analyze the phenotype and outcomes of VLG patients undergoing TAVR. We found that compared with HG and LG, VLG severe AS patients undergoing TAVR (1) have a higher comorbidity burden and worse baseline functional status, (2) may have worse outcomes at 30 days post-TAVR but not at 1 year after adjustment for baseline characteristics, (3) have similar improvements in health status after TAVR, and (4) have lower mean AV gradients at both 30 days and 1 year post-TAVR.

Up to 30% of patients referred for evaluation of AS have LFLG (AV area of ≤1.0 cm^2^ with peak velocity of <4.0 m/s, mean gradient of <40 mmHg, and stroke volume index of ≤35 ml/m^2^), and therefore it is imperative to more clearly understand the phenotype and outcomes (11). Prior studies have shown that patients with classical severe LFLG AS (i.e., with a reduced LVEF) benefit from surgical AVR similar to those with HG AS, though with a significantly increased perioperative hazard (12). However, in most of these prior studies, the LFLG severe AS patients generally have a mean gradient of 30–40 mmHg (3–5, 13–16). A prior analysis of patients with severe AS undergoing TAVR with a self-expanding valve showed that resting pre-TAVR mean gradient was associated with 1-year mortality, but very few patients had a mean gradient of <25 mmHg (17). In addition, the diagnosis of VLG AS is challenging and not well-standardized. The resultant combination of a very symptomatic patient, uncertainty as to whether the AV disease is the culprit for the symptoms, and unknown outcomes of AV intervention presents a challenge for decision-making.

Compared with LG and HG, patients with VLG severe AS had a greater burden of comorbidities including atrial fibrillation/flutter, current dialysis, home oxygen therapy, lower LVEF, and worse mitral and tricuspid regurgitation. The higher prevalence of moderate or severe mitral regurgitation may contribute to the lower mean AV gradients in the VLG group because forward flow may be proportionally reduced (18). Furthermore, the higher prevalence of atrial fibrillation/flutter may also contribute to the lower mean AV gradients due to beat-to-beat variability. While the standard practice is to report the mean AV gradient averaged over several beats when the R–R interval varies (as in atrial fibrillation), recent evidence suggests that this may underestimate the true gradient and therefore the severity of AS (19). Further, VLG patients were more symptomatic at baseline; the median KCCQ-12 summary score was 32.1, and more than 70% had NYHA class III or IV heart failure symptoms. Although this is consistent with our hypothesis that VLG patients are sicker with worse cardiac output resulting in lower AV gradients and worse functional status, selection bias for more symptomatic patients in this group is also noticed. Given the challenges with diagnosis and the uncertain outcomes of TAVR in this population, our Heart Team only recommended TAVR for those patients with VLG AS who were symptomatic, whereas the symptom threshold to recommend TAVR was likely lower in the HG and LG groups.

We found that patients with VLG AS had a higher unadjusted rate of the combined poor outcome (death, KCCQ-12 of <45, or decrease in KCCQ-12 of ≥10) at 30 days and 1 year compared with the LG and HG groups. However, after adjustment for differences in baseline characteristics, VLG patients had a similar rate of the combined poor outcome both at 30 days and 1 year. After multiple imputation for missing data, patients with VLG AS had a higher risk of the combined poor outcome at 30 days but not at 1 year. This is consistent with prior observations of LG patients undergoing SAVR which suggested an overall benefit from SAVR but a higher perioperative hazard compared with HG patients (12). Similarly, VLG patients had a higher mortality rate during follow-up, reaching 23.2% at 1 year compared with 9.1% for the HG group. However, no mortality difference was found between gradient groups after adjustment for baseline differences, and we also did not find a difference in the causes of mortality between groups. It is notable that our observed 1-year mortality in the VLG AS is significantly lower than the 1-year mortality rate of untreated severe symptomatic AS in previous studies (1, 2). Taken together, these observations suggest that while VLG patients have a higher 1-year mortality rate compared with LG and HG severe AS patients, that mortality is largely a function of their comorbidities, and still a likely mortality benefit to the treatment of VLG severe AS patients is reported.

Importantly, our data show that most patients in this study, all of whom were diagnosed with severe AS by our institution's Heart Team, had a significant improvement in symptoms after TAVR regardless of resting AV gradient. VLG patients had the lowest median KCCQ-12 summary score at baseline but at 30 days and 1 year had a median KCCQ-12 summary score equivalent to those of LG and HG patients. Furthermore, more than 90% of VLG patients had at least a 5-point improvement in KCCQ-12 summary score at 30 days and 1 year, a change that has previously been shown to be clinically significant and which is consistent with the improvements seen in the LG and HG patients (6). In addition, the 1-year KCCQ-12 scores in all three gradient groups are similar to those in the most recent STS/ACC TVT Registry report (median of 84.38) (8). Therefore, there is a little doubt that TAVR markedly improved symptoms in the patients in our study regardless of resting AV gradient.

Our mortality and health status outcome findings suggest that patients with severe symptomatic AS benefit from TAVR regardless of their resting transvalvular gradient. However, VLG patients have a considerably elevated post-TAVR mortality rate that is likely a function of their comorbidity burden. Therefore, we suggest that selection of VLG patients for TAVR should be particularly focused on the likelihood of symptomatic improvement with TAVR with a particularly careful assessment of the non-valve-related expected survival. In particular, it is important for the Heart Team to clearly evaluate if the symptoms are truly due to severe AS or another comorbidity. This contrasts with HG severe AS patients in whom the intermediate- to long-term mortality benefit of TAVR is a primary driving force in the decision to pursue intervention.

Perhaps unsurprisingly, we found that patients in the VLG group had lower AV gradients by echocardiography at 30 days and 1 year post-TAVR. This suggests that patients in the VLG group truly have markedly reduced stroke volume at baseline and that stroke volume does not fully normalize after TAVR. This finding implies that post-TAVR gradients should be interpreted in the context of the pre-TAVR gradient in patients with reduced stroke volume; several years after TAVR, a “normal” post-TAVR gradient may in fact indicate recurrent stenosis in a patient with a very low mean AV gradient pre-TAVR. If TAVR utilization expands in this patient group, definitions of “normal” post-TAVR gradients according to valve model and size may need to be updated to account for these considerations. In addition, these definitions may need to rely more upon measures of valve obstruction that are less dependent on transvalvular flow (e.g., effective orifice area).

In our limited analysis comparing mean AV gradient and indexed AV area by echocardiography and invasive assessment, a lack of correlation in the mean gradient was seen. Although some patients invasively had a lower mean gradient, many had a considerably higher gradient. Although invasive hemodynamics were available in only one-third of patients, this finding highlights the importance of invasive evaluation of all patients with high suspicion for AS as the etiology of their symptoms when the echocardiographic measurements are discordant (20). Further analysis with a larger cohort and more complete hemodynamic data is required to further assess this question.

### Study strengths and limitations

Strengths of our study include the use of a composite, patient-centered “poor outcome” that includes both mortality and health status outcomes, the relatively large proportion of VLG patients, and standardized baseline covariates and outcomes. Our study has several limitations. The single-center nature of our study may affect the generalizability of the findings, which require duplication in a larger, more diverse data set. The retrospective design of this study allows determination of association but not causation, and clinical covariates and outcomes were not strictly adjudicated. Importantly, our study lacks data on stroke volume and transvalvular flow, which may be at least as important as the mean gradient. Our study is also limited by suboptimal 1-year follow-up; 61.1% of patients in our study had death or a KCCQ-12 score at 1 year, which is somewhat lower than the 67.2% rate of the same in the most recent STS/ACC TVT Registry report (8). Although we used multiple imputation to address this shortcoming, we cannot exclude differential loss to follow-up leading to bias in our results. For example, sicker patients may be less likely to return for follow-up, and if those patients are more frequent in the VLG group, the VLG group outcomes may look more favorable. More complete and longer-term follow-up data are required to confirm our findings in other cohorts. We also do not have data regarding physical frailty, which may be an important contributor to outcomes in the VLG population, and invasive hemodynamics were available only for a minority of VLG patients. Finally, because we only have follow-up to 1 year, we are unable to exclude differences in outcomes beyond this time point.

## Conclusions

Compared with HG and LG patients with severe AS undergoing TAVR, patients with VLG have a higher burden of comorbidities and symptoms. VLG patients have a higher rate of a poor post-TAVR outcome at 30 days and 1 year, though these differences are not significant after adjustment for baseline characteristics. Similarly, VLG patients undergoing TAVR have a higher mortality rate through 1 year of follow-up though this difference is no longer significant after adjustment for baseline differences between groups. Nearly all patients with VLG severe AS who underwent TAVR had marked improvement in symptoms. Our results suggest that severe symptomatic AS patients benefit from TAVR regardless of baseline mean gradient, though VLG patients primarily benefit in terms of symptomatic improvement rather than increased survival. Further study should improve the diagnosis of VLG severe AS both with and without a preserved LVEF, refine patient selection for valve intervention, and determine long-term outcomes.

## Data Availability

The original contributions presented in the study are included in the article/**Supplementary M**aterials, further inquiries can be directed to the corresponding author.
