# Supplementary material for: Outcomes among patients undergoing transcatheter aortic valve replacement with very low baseline gradients
Source: Front Cardiovasc Med. 2023 Aug 4;10:1194360. doi: 10.3389/fcvm.2023.1194360 (PMC10436597; doi:10.3389/fcvm.2023.1194360)

**Supplemental Figure 1. Kaplan-Meier survival curve estimates of survival without hospital readmission.** VLG = very low gradient; LG = low gradient; HG = high gradient.


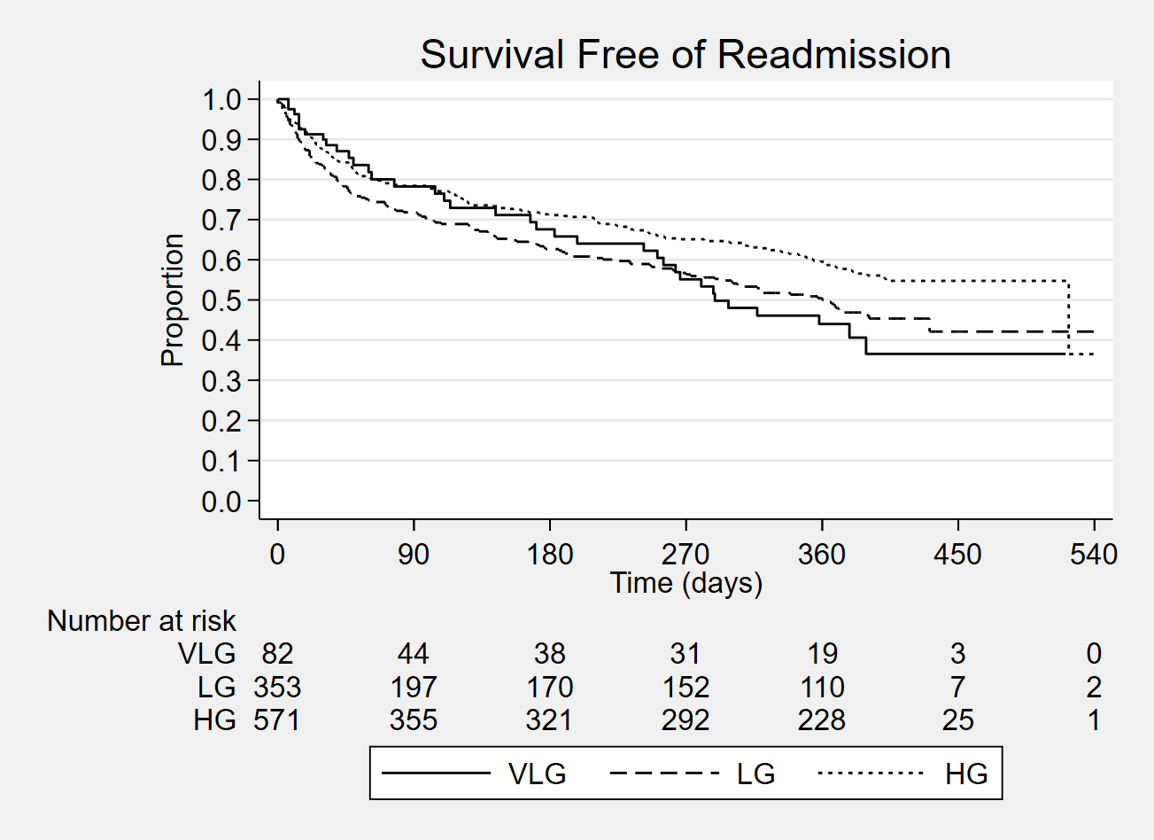


**Supplement**

*Invasive Hemodynamics*

Twenty-nine of 82 (35%) VLG patents had clinically-indicated right and left heart catheterization at our institution before TAVR. Baseline characteristics did not differ between VLG patients without and with RHC except for estimated GFR (48.7 ml/min/1.73 m^2^, IQR 38.3-65.6 ml/min/1.73 m^2^ vs 64.5 ml/min/1.73 m^2^, IQR 43.4-82.1 ml/min/m^2^, respectively; p=0.039) and peak AV velocity (3.2 m/s, IQR 3.0-3.3 m/s vs 2.9 m/s, IQR 2.7-3.1 m/s, respectively, p=0.002). In these 29 patients, TTE-derived mean gradients did not correlate with invasive mean gradient (r=0.27, p=0.16). Similarly, TTE-derived indexed AV area did not correlate with that derived by invasive measurement (r=0.33, p=0.090; **Supplemental Figure 2**). We analyzed the primary combined outcome after re-categorization of severe AS subtype using available invasive mean gradients; thirteen (16%) VLG patients were re-categorized (11 to LG, and 2 to HG). This change in baseline gradient categorization did not significantly affect the primary combined poor outcome at one year (VLG: 50.0%, LG: 29.8%, HG: 23.4%, p=0.001).

**Supplemental Figure 2 –** Correlation in subgroup of patients between transthoracic echocardiogram and invasive hemodynamics


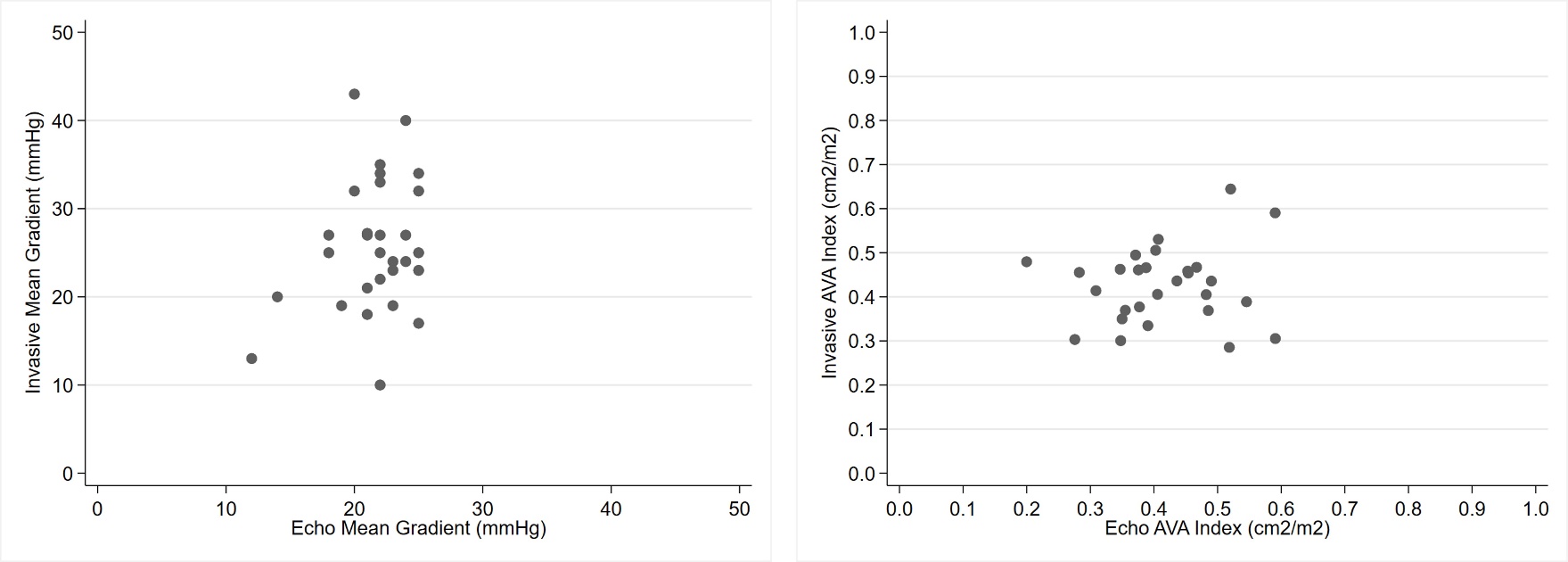

Supplement: Supplementary file 1 [file Datasheet1.docx]
